# Exceeding the limit for microscopic image translation with a deep learning-based unified framework

**DOI:** 10.1093/pnasnexus/pgae133

**Published:** 2024-03-29

**Authors:** Weixing Dai, Ivy H M Wong, Terence T W Wong

**Affiliations:** Department of Chemical and Biological Engineering, Translational and Advanced Bioimaging Laboratory, Hong Kong University of Science and Technology, Hong Kong 999077, China; Department of Chemical and Biological Engineering, Translational and Advanced Bioimaging Laboratory, Hong Kong University of Science and Technology, Hong Kong 999077, China; Department of Chemical and Biological Engineering, Translational and Advanced Bioimaging Laboratory, Hong Kong University of Science and Technology, Hong Kong 999077, China

**Keywords:** microscopic image translation, deep learning, supervised learning, unsupervised learning, unified framework

## Abstract

Deep learning algorithms have been widely used in microscopic image translation. The corresponding data-driven models can be trained by supervised or unsupervised learning depending on the availability of paired data. However, general cases are where the data are only roughly paired such that supervised learning could be invalid due to data unalignment, and unsupervised learning would be less ideal as the roughly paired information is not utilized. In this work, we propose a unified framework (U-Frame) that unifies supervised and unsupervised learning by introducing a tolerance size that can be adjusted automatically according to the degree of data misalignment. Together with the implementation of a global sampling rule, we demonstrate that U-Frame consistently outperforms both supervised and unsupervised learning in all levels of data misalignments (even for perfectly aligned image pairs) in a myriad of image translation applications, including pseudo-optical sectioning, virtual histological staining (with clinical evaluations for cancer diagnosis), improvement of signal-to-noise ratio or resolution, and prediction of fluorescent labels, potentially serving as new standard for image translation.

**Significance Statement**

In deep learning-based microscopic image translation, data are usually only roughly paired such that supervised learning could be invalid due to data unalignment, and unsupervised learning would be less ideal as the roughly paired information is not utilized. This study proposed a unified framework for dealing with the general cases of roughly paired data and meanwhile creating a theoretical linkage between supervised and unsupervised learning. The superiority of the unified framework was validated in a myriad of image translation applications, including pseudo-optical sectioning, virtual histological staining, improvement of signal-to-noise ratio or resolution, and prediction of fluorescent labels, both qualitatively and quantitatively with clinical evaluations for cancer diagnosis.

## Introduction

Facilitated by recent advancements in deep generative networks (1–6), deep learning-based image translation in optical microscopy has drawn increasing traction due to its superior performance in various applications. For example, virtual histological staining computationally transforms label-free images into histochemically stained images, which can be directly analyzed by pathologists without any chemical reagents, saving time and cost for sample preparation (7–11). High-quality microscopic images can be computationally restored with deep generative networks from low signal-to-noise ratio (SNR) or resolution so that the range of observable biological phenomena is extended (12–16). Furthermore, computational models can also be used to predict fluorescent labels from transmitted-light images of unlabeled fixed or live biological samples to avoid the limitations of physical fluorescent labeling such as spectral overlap and perturbations of the experiment (17).

According to the availability of paired training data, deep learning models for image translation can be divided into supervised learning and unsupervised learning. For paired data, supervised learning can generate state-of-the-art results due to their strong ability in modeling complex features with high accuracy (10, 18–22). Unsupervised learning is capable of learning with unpaired image data which are easy to be acquired (23–28). Although shadowed by the successes of supervised learning, unsupervised learning is equally important in the long term as paired data are often not available, and meanwhile, the learning mode of humans and animals is largely unsupervised (29). Except for purely convolutional neural network-based networks such as CARE (16), which performs well on a wide range of image restoration tasks, many existing deep generative models for image translation are based on generative adversarial networks (GANs). For example, pix2pix (22) learns the mapping from a source domain to a target domain by conditional GANs and has been widely used in image translation tasks. CycleGAN translates unpaired images from a source domain to a target domain by introducing a cycle-consistency loss (28). Diffusion models, which are inspired by nonequilibrium thermodynamics (30), have recently demonstrated their superior results on various image processing tasks (31–35). With additional conditioning of source images (33), diffusion models serve as a powerful tool for image translation with paired datasets.

Prior work on deep generative networks for image translation has been limited to purely supervised learning or unsupervised learning, without investigating their relationship. Nevertheless, fully paired data and unpaired data are the two extreme cases and generally, there are many intermediate states where the data are roughly paired. For example, comparing the images of widefield microscopy and confocal microscopy with optical sectioning, the same sample shares similar information under the two imaging modalities. However, the information is not identical as only an optically selected layer is provided in confocal images, while the integration of all the layers is provided in widefield images. As their detailed structures are different at a pixel level, they are only roughly paired and unsuitable to be trained in a supervised manner. In addition, even for fully paired images, there can be some unpaired parts and local deformation due to tissue distortion, shrinkage, folding, and artifacts generated during sample preparation (36, 37). Therefore, fully paired and unpaired data can be regarded as two special forms of the general data type. Similarly, such examples can also be found in virtual histological staining, in silico fluorescent labeling, and image restoration from low SNR or resolution. In these circumstances, it can be confusing to choose the training models because the performance of supervised learning could be limited due to image unalignment, whereas if we train with unsupervised learning, the results would be still unsatisfactory because the roughly paired information in the data is not fully utilized. Therefore, both purely supervised and unsupervised learning are not the optimal solutions in these conditions.

Several recent studies have been conducted to improve image translation, as well as other visual tasks, at a patch level (22, 38–41). For example, when conditional GANs are used for image translation, PatchGANs with moderate patch size in discriminator outperform PixelGAN and ImageGANS with whole input size (22). Applying conditional GANs hierarchically across multiple scales generates visually appealing results for synthesizing high-resolution images (41). Contrastive learning focusing on the heterogeneous semantics between the image patches enhances the spatial correspondence for image translation (42). However, the patches defined in these papers are all subsets of the input image to the network and they only reflect the information in the input image, rather than the regional information for the whole microscopic image. Microscopic images are usually in high resolution and if they are directly cropped as input of the network, much regional information would be lost. Therefore, including such regional information could be crucial for high-performance microscopic image translation.

There is a clear need for establishing a learning method that does not assume the data to be fully paired and meanwhile can learn effectively from the regional information depending on the degree of data alignment such that the training model, ideally, contains only the advantages of both unsupervised and supervised learning. In this regard, we propose a unified framework (U-Frame) for modeling all types of data, including fully paired, roughly paired, and unpaired data. The proposed U-Frame consists of a special GAN and can be fitted to data with any degree of image alignment by an automatically adjusted hyperparameter. The testing results on different applications show U-Frame's superiority over both supervised and unsupervised learning even for fully registered image pairs. U-Frame provides a unified solution for various kinds of image data, potentially serving as a new standard for achieving high-quality microscopic image translation.

## Results

### Principle of U-Frame

**Fig. 1.**
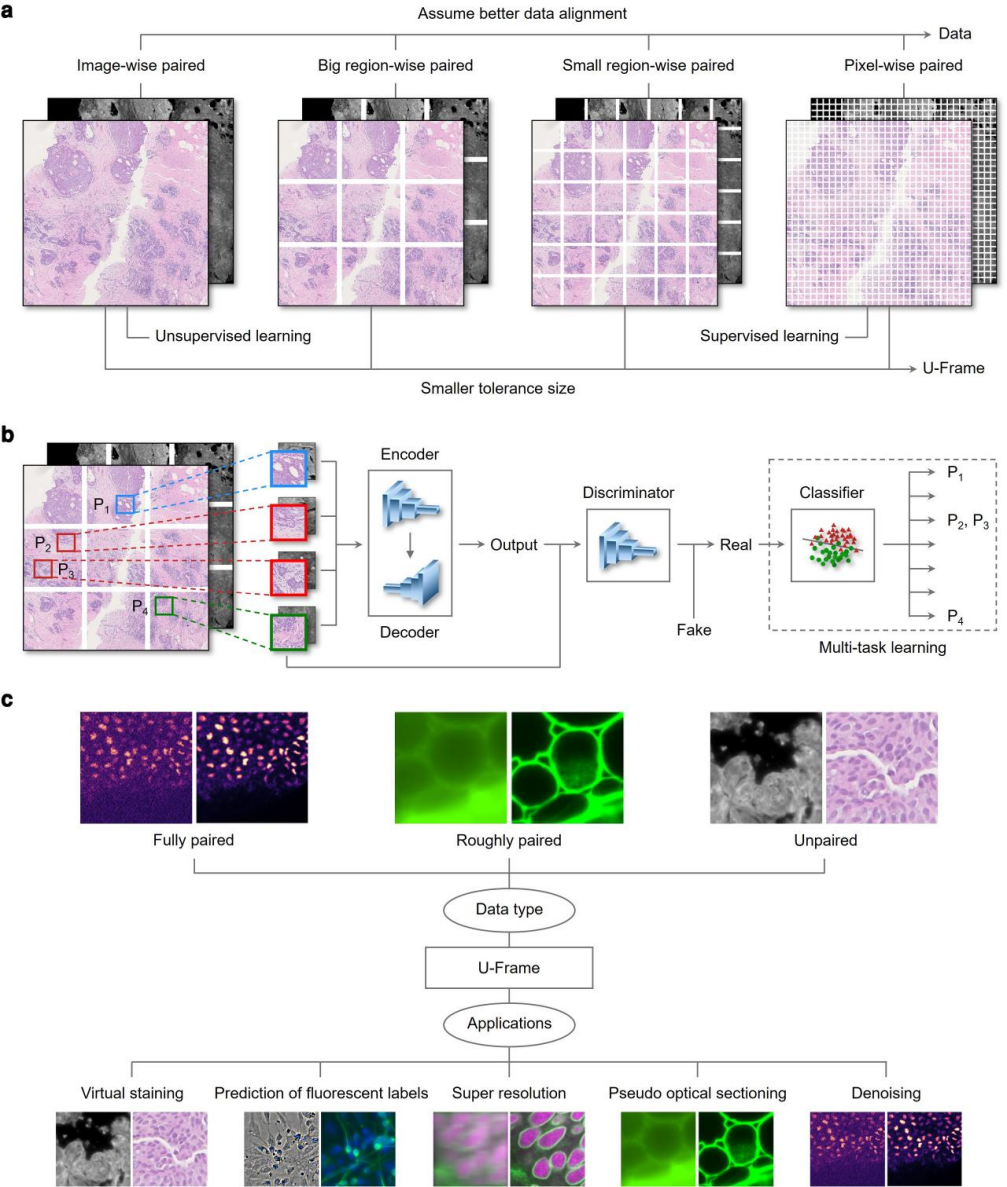
Overview of U-Frame. a) Working principle. U-Frame bridges supervised and unsupervised learning in terms of data alignment. U-Frame can be biased to either unsupervised or supervised learning by adjusting the parameter controlling the size of the region, i.e. the tolerance size. b) U-Frame's architecture. Multitask learning is used for the training of U-Frame. The architecture is GAN-based, where the discriminator is sensitive to the location of input regions. $P_{1}$ and $P_{4}$ are from different regions and classified as different classes in the discriminator, while $P_{2}$ and $P_{3}$ are from the same region. Therefore, they are classified as the same class in the discriminator. c) The types of data and applications that can be handled by U-Frame.

We divide the data of microscopic image translation from a source domain to a target domain into three conditions: pixelwise paired, imagewise paired, and regionwise paired. A source image and a target image are pixelwise paired if the pixels from the same position can be aligned and represent the same physical object. Imagewise paired refers to the condition that the whole source image and target image represent the same physical object, but are not necessary to be aligned in any specific region or pixel. Regionwise paired refers to the condition between the pixelwise paired and imagewise paired, where a source image and a target image can be aligned in any region with a specific overlapping area, rather than pixel or the whole image. For unsupervised image translation, images from a source domain are transformed into a target domain of a different style, where the feature patterns are assumed to be consistent in a domain. Therefore, the training data for unsupervised learning can be regarded as imagewise paired. However, microscopic images are more complex and the feature patterns are usually inconsistent in a domain. For example, the cells and connective tissues in the cancer regions and normal regions shown in a microscopic image are different in terms of shape, size, and structure. In this case, supervised learning is a better choice for modeling the detailed structural information if pixelwise paired data are available. However, the acquisition of pixelwise paired data is time-consuming and sometimes impossible. A more general case would be that the image data are in an intermediate state (e.g. regionwise paired). Therefore, the image data are not reaching the level of pixelwise paired, which, however, are not just imagewise paired. To fully utilize the regionwise paired information depending on the degree of image misalignment, U-Frame establishes a unified framework that bridges supervised and unsupervised learning (Fig. 1a).

In U-Frame, the feature patterns in an image of a specific domain are assumed to be inconsistent in different regions. To simplify the problem, the image is divided into multiple mutually exclusive regions of the same size, which is defined as tolerance size. In each region of tolerance size, the feature pattern is assumed to be consistent and transformed in an unsupervised manner so that pixelwise registration is not required. Therefore, U-Frame with a large tolerance size is biased toward unsupervised learning, while U-Frame with a small tolerance size is biased toward supervised learning.

Multitask learning is used in U-Frame for modeling the networks of different regions (Fig. 1b). To reduce the risk of overfitting, all the networks of different regions share the same encoder, decoder, and discriminator, except for the last layer of the discriminator. There are two unique nodes for each region in the last layer of discriminators, where one of the nodes represents its real class information and is to be trained for better exploration of the details in the training images, while the other node represents fake generated information. All the nodes representing fake generated information are combined into one node. Therefore, there are total *N* + 1 nodes in the last layer of the discriminators, where *N* is the number of regions in the training dataset. The hyperparameter, tolerance size, influences the network by controlling the number of neural nodes in the unique layer during training, which corresponds to the number of regions.

The tolerance size is optimized and selected by an automatic strategy (Table 1) for the training of U-Frame. Specifically, for a potential tolerance size, corresponding regions are randomly cropped from the source and target images, which are then imported to contrastive language-image pretraining (CLIP) to generate a compressed feature representation of the input region (43). Pearson correlation of cropped regions from the source and target images, which is calculated based on the feature representation, is used as the objective function for optimization. The assumption in U-Frame is that the source and target images share similar feature patterns in the same region; therefore, we optimize the tolerance size by maximizing the objective function within a defined range.

**Table 1.** Pseudocode of the algorithm for optimizing the tolerance size.

Algorithm: automatic optimization of tolerance size for U-Frame
def tolerance_optimization (source_image, target_image, lower_bound, upper_bound):
objective_optimal = 0
for tol in arange (lower_bound, upper_bound, step):
objective = 0
for i in range (num_sampling):
patch_source = random_crop (source_image, tol)
patch_target = random_crop (target_image, tol)
feature_source = CLIP (patch_source)
feature_target = CLIP (patch_target) objective += pearson_correlation (feature_source, feature_target) if objective > objective_optimal:
tolerance_optimal = tol objective_optimal = objective return tolerance_optimal

The architecture of the proposed framework is shown in Fig. 1b. As shown in Fig. 1b, the input patches P1 and P4 are in different regions and assumed to be inconsistent with each other, while P2 and P3 are in the same region and thereby are regarded as the same class in the final classifier. The architecture of U-Frame is an extension of basic GAN with the architecture of a convolutional neural network. First, the raw image is divided into *N* regions according to their spatial sequence, and each region is assigned a class number. In each iteration, a region is selected according to a global sampling rule (Fig. S1) and then expanded and overlaps with its neighboring regions so that features are partially shared among the regions, and patches of fixed size are randomly cropped from the selected region as the input fed into the encoders and decoders of the neural network to obtain the outputs. Next, the outputs are imported into the discriminators, which not only classify generated examples from generators as real or fake classes but also differentiate patches from different regions in the real classes. Specifically, the discriminators identify *N* + 1 classes, including *N* classes for exclusive regions and an additional class for fake generated patches, so that the model can simultaneously adapt to multiple feature patterns in different regions of the image and achieve high accuracy in complex situations. The network structures of encoders, decoders, and discriminators are shown in Fig. S2 and Tables S1–S3.

U-Frame is capable of handling various types of data including fully paired, unpaired, and roughly paired data. It is particularly suitable for handling roughly paired data where both supervised learning and unsupervised learning may be undesired. We demonstrate that U-Frame shows superior performance and consistently outperforms both supervised and unsupervised learning in various applications such as image translation from widefield to confocal images, prediction of fluorescent labels, virtual histological staining, superresolution, and denoising (Fig. 1c). We use pix2pix and conditional diffusion model as supervised learning methods, and CycleGAN as the unsupervised learning method to compare performance with U-Frame. Both pix2pix and CycleGAN have the same encoder and decoder as U-Frame. We also include CARE for comparison in the image restoration tasks, where CARE was proposed for image restoration together with the release of related datasets (16).

### Image translation from traditional widefield images to confocal images

Confocal fluorescence microscopy is a popular imaging modality for basic life science study. On optically thick specimens, confocal fluorescence microscopy (44–46) provides superior image quality than that of traditional widefield microscopy by using a pinhole to remove the out-of-focus light originating from layers other than the single focal plane, thus, providing an improved image contrast with the optical sectioning capability. Multiple depth-resolved images of an optically thick specimen can be acquired using confocal fluorescence microscopy. However, the use of a pinhole also implies the requirement of using a sensitive detector or a long exposure time for a high-quality image, leading to a high equipment cost and a high risk of photobleaching (47). Here, we demonstrate the use of U-Frame to digitally transform a widefield image into confocal images with multiple layers, which can share the advantages of both modalities, providing improved image quality and depth-resolved information while not requiring long image acquisition time due to the point-scanning mechanism of a confocal microscope. Furthermore, we could also enjoy the simplistic optical design of a widefield microscope.

**Fig. 2.**
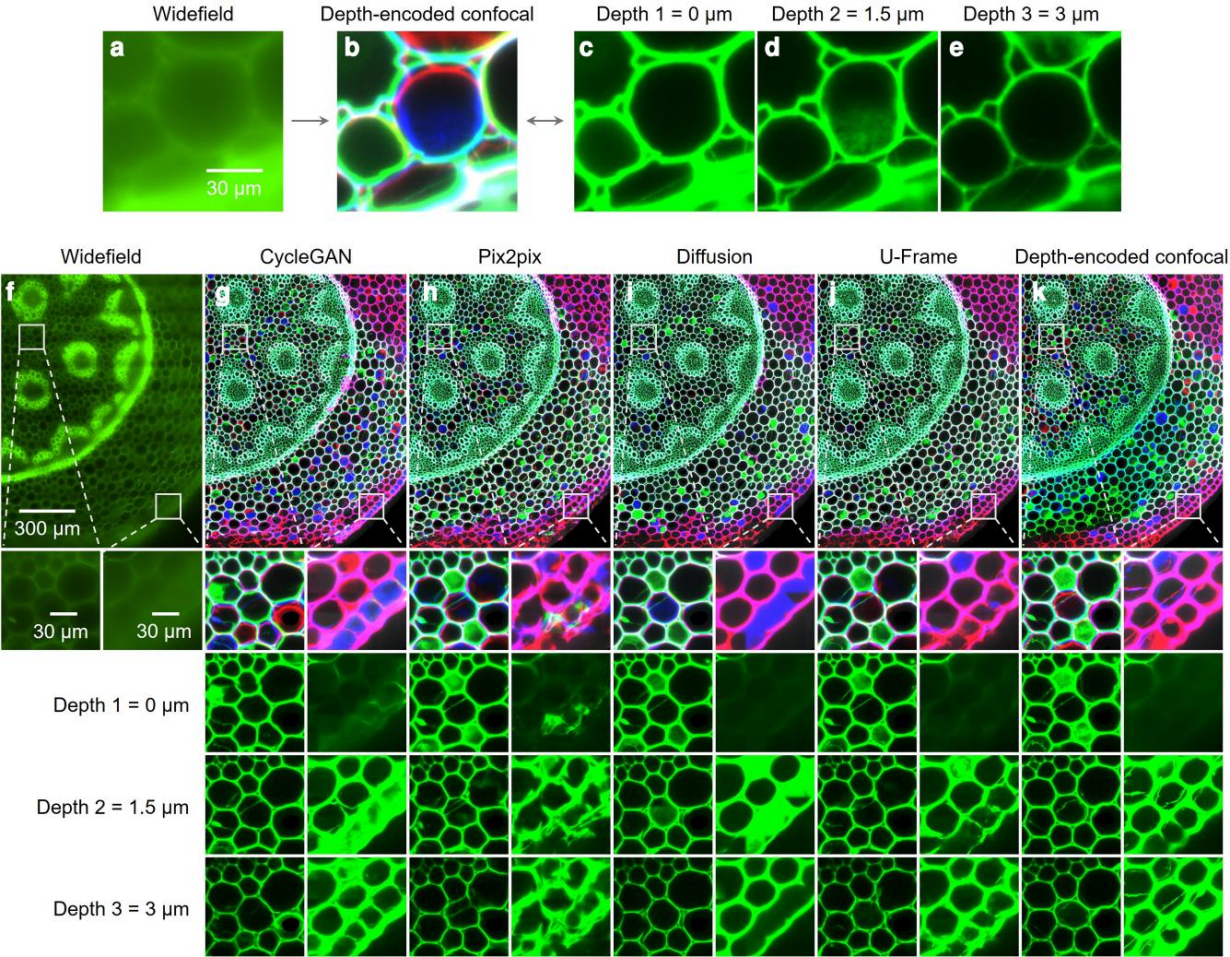
Comparison of deep learning-based image transformations from widefield images to confocal images. a) Widefield image of a plant cell sample. b) Depth-encoded confocal image of A with three different layers. c–e) Corresponding confocal images at the three different depths. f) Widefield image in the test set. g–j) Transformed images from F by CycleGAN, pix2pix, diffusion model, and U-Frame, respectively. k) The corresponding confocal image of F with three different layers.

There are also related works employing deep learning methods to achieve pseudo-optical sectioning images in recent years, including the use of convolutional neural networks and supervised learning to reconstruct optically sectioned images using widefield images as input and trained with spatially aligned structured illumination microscopy images (48, 49). However, it is challenging to transform from widefield images to multiple confocal images with pseudo-optical sectioning capability when the image features vary from layer to layer using a supervised method. In this case, a widefield image (Fig. 2a) and the confocal images from different layers can only be roughly paired for training (Fig. 2b–e), which does not conform to the assumption of pixelwise paired data for supervised learning. For demonstration, here, the confocal images were acquired at three different depths, each 1.5 μm apart (Fig. 2c–e). The depth-encoded confocal image (Fig. 2b) overlays all information at different depths with different colors. The imperfect of supervised learning is validated by the results obtained from the cross-section of a vascular plant stained with safranin to visualize the plant cell wall, and the multiple-layer images transformed by U-Frame were the closest to the ground truth among all the methods, with correct cell wall structure and fluorescence intensity distribution (Fig. 2f–k). The performance of U-Frame for transforming from a widefield image to a single-layer confocal image was also validated (Fig. S3).

### Style transfer from autofluorescence images to histochemically stained images

Histological images of cancer tissue are usually composed of complex morphology with different types of cell and tissue structures. Clinically, hematoxylin and eosin (H&E) stain is the most frequently used routine stain which can provide rich information for visualizing nuclear and cytoplasmic components, and are widely used to diagnose cancer. However, conventional sample preparation involves time-consuming and laborious steps. Therefore, many researchers in the field of biomedical engineering are spending their effort on generating the standard H&E-stained images from the images acquired by a label-free imaging method (e.g. autofluorescence imaging) through virtual staining enabled by deep learning (7–11).

To show that U-Frame is a perfect solution for this application, we first acquired two sets of autofluorescence and H&E-stained images, denoted as dataset I and dataset II, from a human breast biopsy tissue. In dataset I, the source images and target images are from the same tissue slice so that they can be well aligned, while in dataset II, they are from the adjacent tissue slice and can only be roughly aligned. Subsequently, we performed virtual histochemical staining to transform the stain-free autofluorescence images into histochemically stained images.

**Fig. 3.**
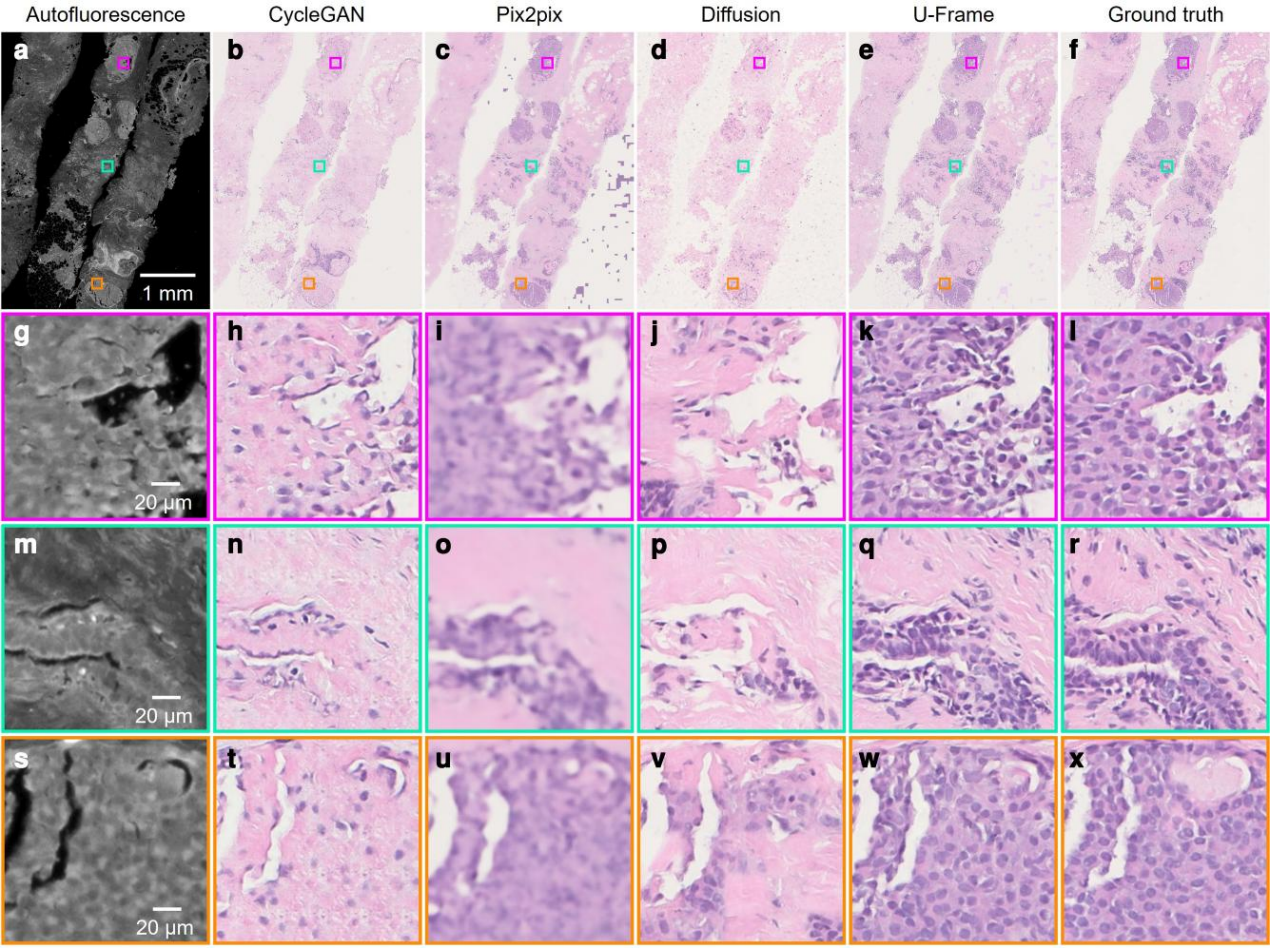
H&E virtual staining of breast cancer tissue (dataset II). In this dataset, autofluorescence and H&E images are from adjacent tissue slices so that they can only be roughly aligned. a) Autofluorescence image to be transformed. b–e) Image transformed by CycleGAN, pix2pix, diffusion model, and U-Frame, respectively. f) Ground truth image of A. g–x) Zoomed-in regions in a–f, respectively.

U-Frame can transform the autofluorescence image into a virtual H&E-stained image with high-quality tissue and cellular distributions (dataset I, Fig. S4), For dataset II, where data are only roughly paired, U-Frame shows its superiority compared to both supervised and unsupervised learning (Fig. 3). Here, the CycleGAN output misses most of the cell nuclei (Fig. 3b, h, n, t), whereas the pix2pix output is greatly blurred such that it is difficult to observe the detailed nuclear information (Fig. 3c, i, o, u). The diffusion model output is clear, but also misses most of the cell nuclei (Fig. 3d, j, p, v). U-Frame can still output nuclear size and shape correctly (Fig. 3e, k, q, w), which can be validated by the H&E-stained images of the adjacent tissue slice (Fig. 3f, l, r, x).

We also demonstrated that U-Frame can also apply to other types of staining. Masson's trichrome was selected as an example due to its wide usage, which is a special stain used clinically for visualizing type I collagen fiber. We validated the high performance of U-Frame using human liver cancer tissue with serious fibrosis, where type I collagen fibers are prominent. The results indicated that U-Frame preserves the nuclear content well and achieved a more accurate color transformation when compared with both unsupervised and supervised learning (Fig. S5).

We further tested the performance of U-Frame on training datasets with two different levels of spatial misalignment of the input data, where supervised methods would be invalid as the data for training are poorly aligned. The first dataset (denoted by S_1_) was generated from H&E virtual staining of human breast cancer images by global affine mapping (50) without local registration so that the data were roughly aligned with slight mismatching. Based on the first dataset, the second dataset (denoted by S_2_) was generated by randomly pairing the patches of autofluorescence and ground truth images where the Euclidean distance is 256 pixels so that the data were poorly aligned. We tested the performance of U-Frame and compared it with CycleGAN on an adjacent tissue section. The results show that the virtual H&E-stained image by U-Frame (Fig. S6) is with higher similarity to the ground truth for both datasets when compared with CycleGAN, which demonstrates the robustness of U-Frame.

### Improvement of SNR or resolution

**Fig. 4.**
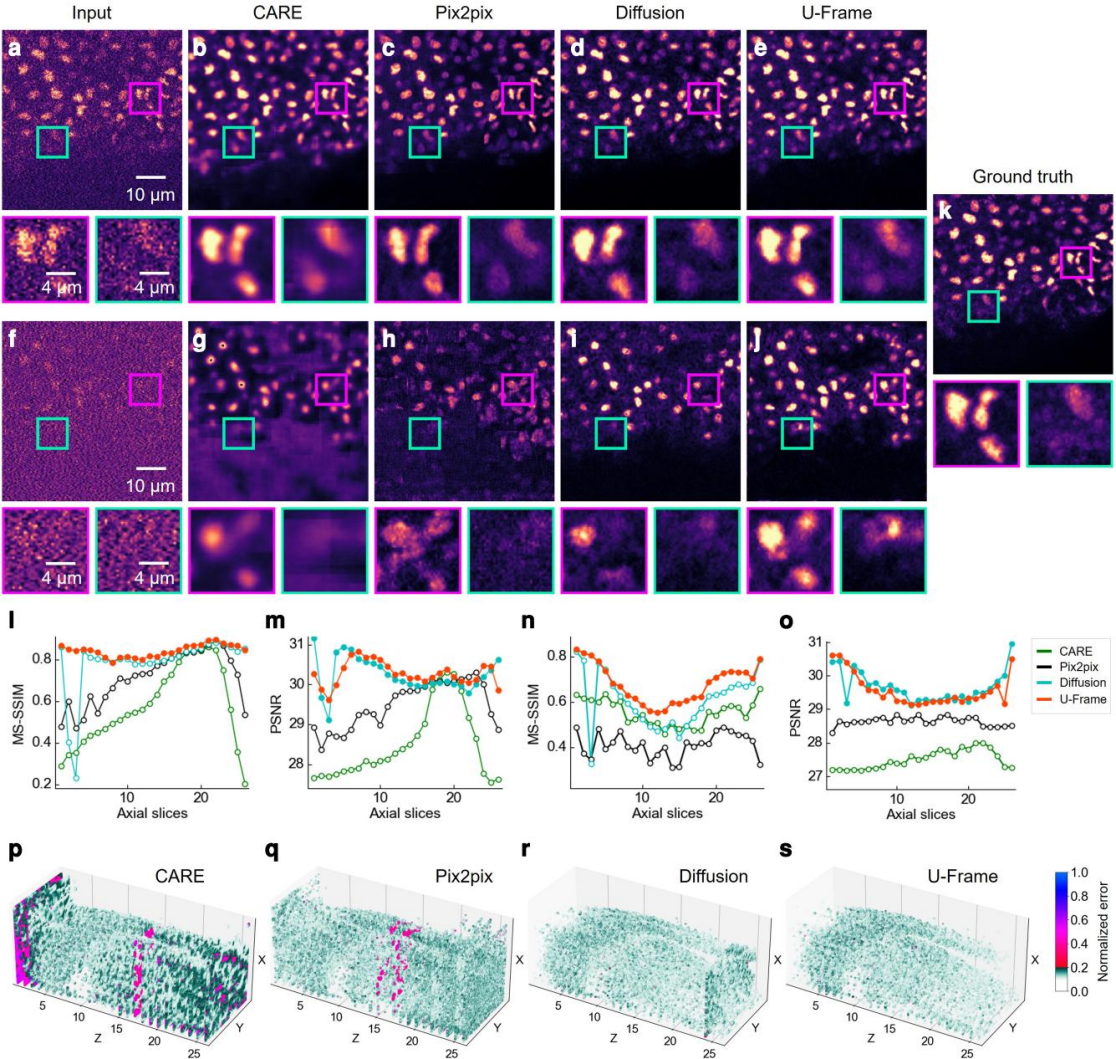
Low-SNR image restoration of *S. mediterranea* (planaria). U-Frame is compared with other fully supervised methods (CARE, pix2pix, and diffusion model) for two levels of low-SNR image restoration. a–e) First level low-SNR image restoration of acquisition with reduced laser power of 0.12 mW and shortened exposure time of 20 ms. The representative images are from the axial slice of 21. f–j) Second level low-SNR image restoration of acquisition with further reduced laser power of 0.05 mW and further shortened exposure time of 10 ms. k) Ground truth (an acquisition with laser power of 2.31 mW and exposure time of 30 ms). l and m) Quantification metrics (MS-SSIM and PSNR) of all the axial slices for the first level low-SNR image restoration. n and o) Quantification metrics (MS-SSIM and PSNR) of all the axial slices for the second level low-SNR image restoration. The hollow dots refer to the metrics of the corresponding method that are smaller than that of U-Frame (*P*-value < 0.05). p–s) Error maps of the volumetric stacks for the second level low-SNR image restoration by CARE, pix2pix, diffusion model, and U-Frame, respectively. The normalized error is the absolute difference between the normalized pixel values of the testing image and the ground truth. The values with differences < 0.1 are masked with white color.

First, we tested for low-SNR image restoration of *Schmidtea mediterranea* (planaria). The images for training and testing are from samples of nucleus-labeled *S. mediterranea* at different developmental stages (16). Alignment of these training images is naturally ensured by interleaving the different imaging conditions during data acquisition, thus, no further image registration is required for supervised learning. We compared the performance of U-Frame with other commonly used supervised methods, including CARE (16), pix2pix (22), and the diffusion model, under two levels of low-SNR acquisitions with reduced laser power and shortened exposure time. Here, the first level is with a laser power of 0.12 mW and an exposure time of 20 ms, while the second level is with a laser power of 0.05 mW and an exposure time of 10 ms (the SNR of the second level is ∼30% compared to that of the first level). For the first level low-SNR acquisition, the overall image qualities restored by CARE (Fig. 4b), pix2pix (Fig. 4c), the diffusion model (Fig. 4d), and U-Frame (Fig. 4e) are all comparable and close to ground truth (Fig. 4k). However, in both the strong signal zoomed-in region and the weak signal zoomed-in region (inset of Fig. 4a), the shape of nuclei and their surroundings restored by U-Frame (inset of Fig. 4e) is more similar to the ground truth when compared with that by CARE (inset of Fig. 4b), pix2pix (inset of Fig. 4c), and the diffusion model (inset of Fig. 4d). Multiscale structural similarity (MS-SSIM) (51) and peak signal-to-noise ratio (PSNR) are calculated to quantify the results. The quantitative metrics of the first level low-SNR acquisition are shown in Fig. 4l and m. The advantage of U-Frame can also be observed in a 3D normalized error plot across all the axial slices (Fig. 4p–s). For the second level low-SNR acquisition, where the input image is more complex as the signals from the features and background are similar, the advantage of U-Frame for all the axial slices is more significant compared with the first level low-SNR acquisition (Fig. 4f–j, n, o). The results indicate that U-Frame consistently outperforms supervised methods even for perfectly aligned image pairs, and this holds especially for complex cases.

We also applied U-Frame to improve image resolution for two-channel image acquisitions of the developing eye of zebrafish (*Danio rerio*) embryos. The two channels were concatenated as the input of the network for training. The results show that both channels of nuclei and the nuclear envelope were well recovered from the original anisotropic images by U-Frame (Fig. S7). Meanwhile, different shapes of nuclei can be clearly identified with the help of collaborative effect from transformed nuclei and nuclear envelope, indicating the capability of U-Frame for multicolor complex image restoration.

### Prediction of fluorescent labels from unlabeled images

**Fig. 5.**
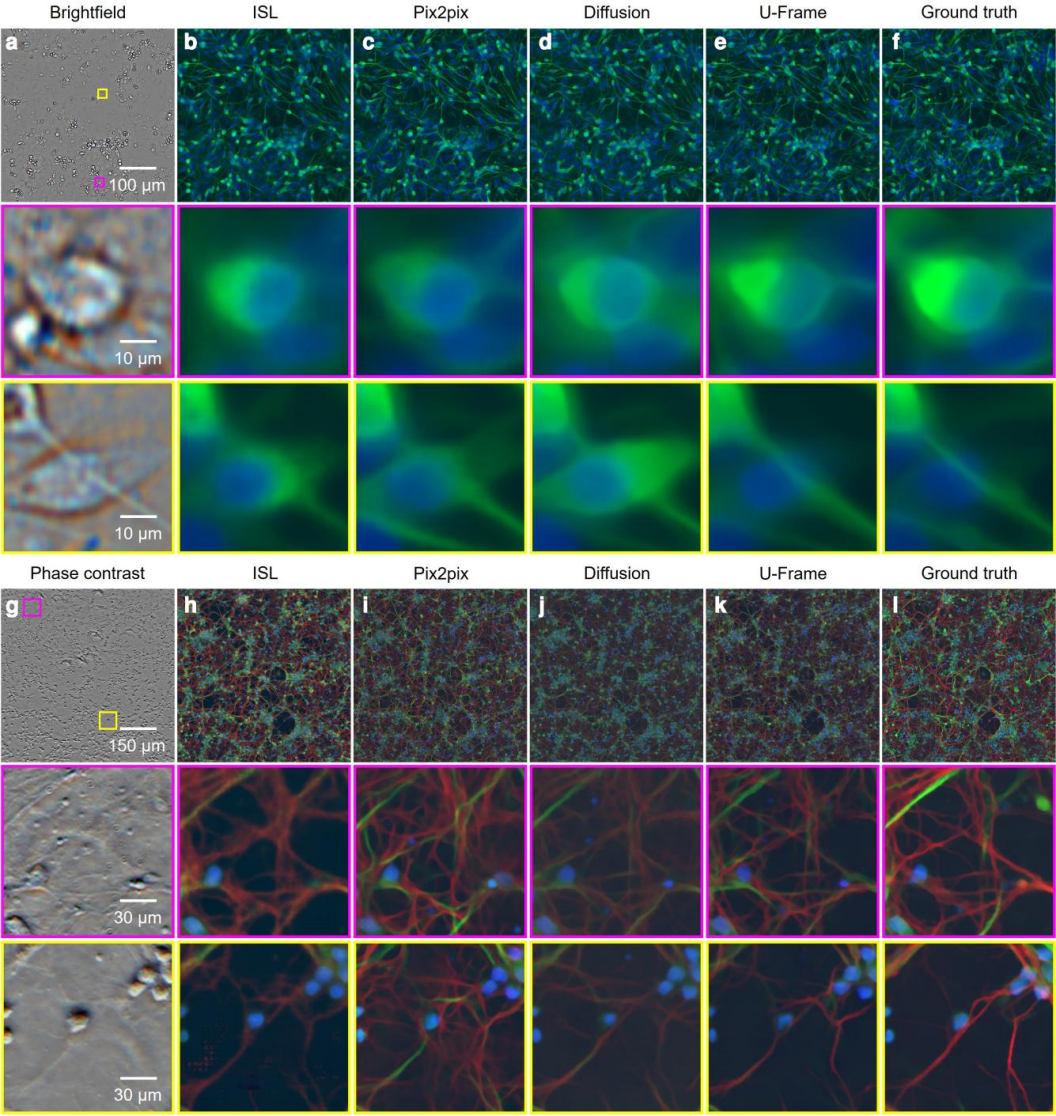
Predicting fluorescent labels from unlabeled images. a) The brightfield images that are used for predicting the fluorescent labels of neurons. b–e) Predicted neuron labels from A by ISL, pix2pix, diffusion model, and U-Frame, respectively. f) Real neuron labels of A as the ground truth. g) Phase-contrast images that are used for predicting the fluorescent labels of axons. h–k) Predicted axon labels from G by ISL, pix2pix, diffusion model, and U-Frame, respectively. l) Real axon labels of G as the ground truth. The colored squares show zoomed-in views of the corresponding marked subregions.

We further tested U-Frame with two open-source datasets (17) for predicting fluorescent labels from unlabeled images (Fig. 5). In the first dataset, the brightfield images are used as the input to predict whether a cell is a human motor neuron by in silico labeling (ISL) (17), pix2pix, diffusion model, and U-Frame (Fig. 5a–f). In the second dataset, multicolor axon labeling is predicted from phase-contrast images (Fig. 5g–l). In both of the datasets, U-Frame is superior in showing the details at a cellular level with higher accuracy and contrast compared with other methods (Fig. 5).

### Clinical value of U-Frame for cancer diagnosis

**Fig. 6.**
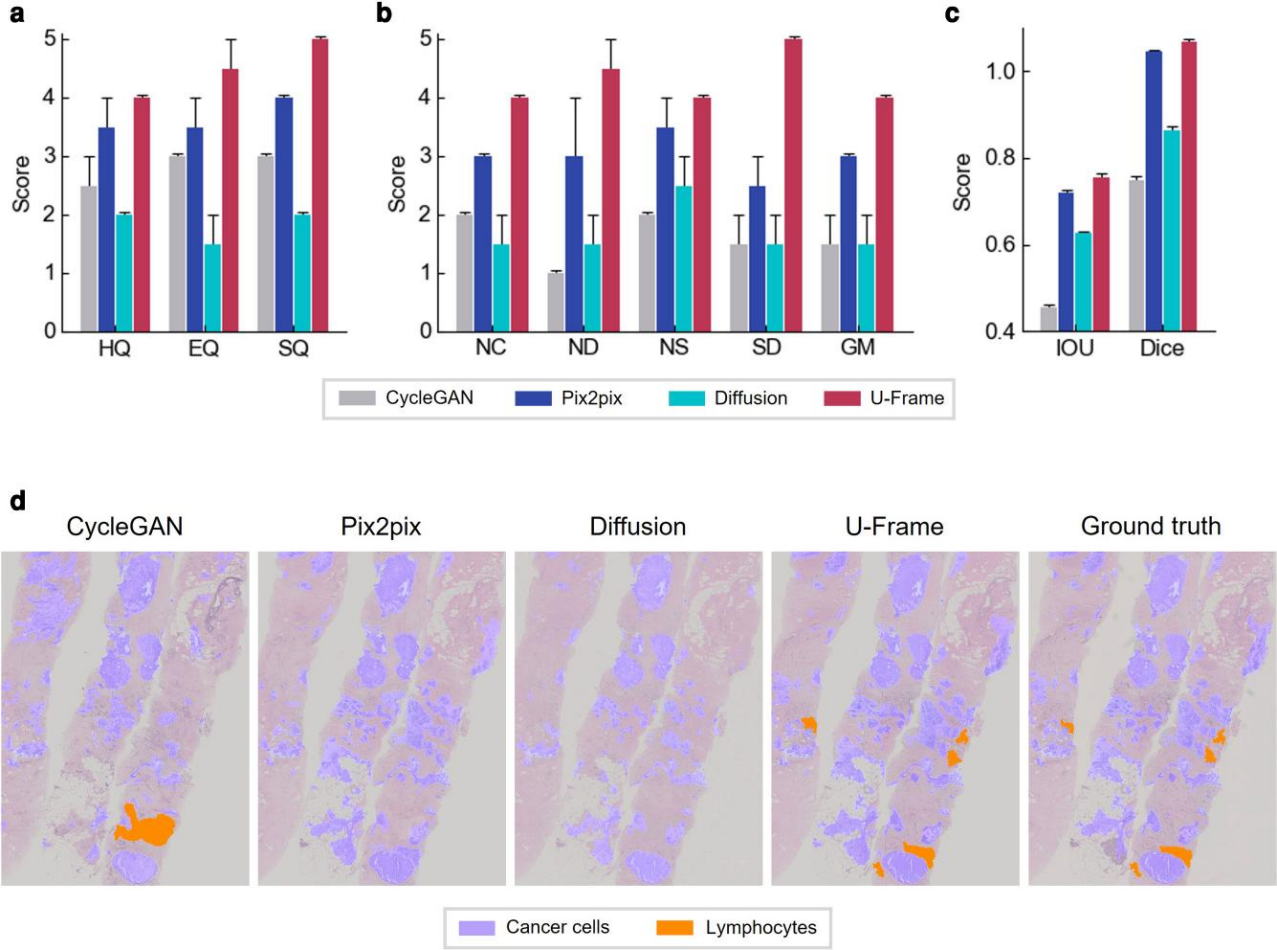
Blinded evaluation of virtually stained images by histological experts. a) Evaluation in terms of stain quality with evaluation parameters of hematoxylin quality (HQ), eosin quality (EQ), and overall stain quality (SQ). The meaning of the score is as follows: 5, excellent; 4, very good; 3, good; 2, acceptable; 1, unacceptable; 0, cannot show/cannot determine. b) Evaluation in terms of morphology quality with evaluation parameters of nuclear circularity (NC), nuclear density (ND), nuclear size (NS), stroma details (SD), and GM. The meaning of the score is the same as the evaluation of stain quality. c) Evaluation in terms of cell-type classification with parameters of IOU and Dice score. IOU and Dice scores were calculated based on the binary mask of cancer cells. d) Matching cancer cells and lymphocytes for virtually stained images and ground truth.

After evaluating U-Frame on multiple applications of image translation, we passed the histochemically stained images of the human breast biopsy tissue and the corresponding images generated by different virtual staining approaches to two histopathology experts to show the clinical impact of U-Frame for cancer diagnosis by blinded evaluation. The pathologists were blinded to virtual staining approaches and asked to give scores indicating the quality of virtually stained images for cancer diagnosis. The quality of virtually stained images was evaluated in three aspects: (i) the stain quality which describes the color information consistency with the ground truth; (ii) the morphology quality which describes the structural consistency with the ground truth; and (iii) a cell-type classification that evaluates the impact of image interpretation affected by different performance of virtual staining. As shown in Fig. 6, U-Frame has the highest scores compared to CycleGAN, pix2pix, and the diffusion model in terms of stain quality (Fig. 6a), morphology quality (Fig. 6b), and cell-type classification (Fig. 6c and d). The results of our clinical evaluation indicate that pathologists can recognize more histopathological features in virtually stained images by U-Frame than that by other deep learning approaches.

### Validation of the strategy for automatically selecting tolerance size

We validated the strategy for automatic optimization of tolerance size on the poorly aligned breast cancer dataset (S_2_) where a random shift of 256 pixels was introduced. We recorded the objective function during optimization (Fig. S8A), and then validated the performance of transformed images for five different tolerance sizes (Fig. S8B and C). From the curve of the objective function, we can see that as the tolerance size increases, the objective function remains at a high level before it reaches the maximum when the tolerance size is 1,024, and then drops dramatically when the tolerance size is larger than 2,048. Both the quantitative metrics of MS-SSIM and PSNR (Fig. S8B) confirm this trend and reach their maximum when the tolerance size is 1,024. The trend can also be observed from the quality of transformed images, where the quality of virtually generated nuclei remains high at the tolerance of 256 and 512, reaches best at 1,024, begins to drop at 2,048, and then drops dramatically at 4,096 (Fig. S8C). The results indicate that the performance of U-Frame is not too sensitive for tolerance size near the optimal value, which allows us to attain the best performance safely.

## Discussion

We have introduced a unified framework, U-Frame, to serve as a bridge for unsupervised and supervised learning for microscopic image translation. U-Frame is a general solution for microscopic image translation and could be applied to various kinds of imaging data without requiring to be pixelwise paired. We validated that the transformed images by U-Frame are of high quality, which outperforms unsupervised methods for roughly paired or unpaired data, and even supervised methods for fully paired data.

Supervised learning is usually regarded as the state-of-the-art method when paired data are available. However, our study shows that even with pixelwise paired data, U-Frame still consistently outperforms supervised learning. We speculate that the robustness of U-Frame is attributed to its strong ability to balance the model complexity by training with regionwise paired data, where it performs in an unsupervised manner for images from the same region and meanwhile supervision is done among the regions in the discriminators. Especially for datasets that are not well paired, the images generated by a fully supervised method with pixelwise supervision such as pix2pix can be blurred (Fig. 4). The reason is that in such case, when pixelwise supervision is forced on the poorly paired data, more feature patterns need to be learned for full feature representation of the dataset. As a result, the network is forced to increase capacity for the increased feature patterns and the variance of the network becomes large. The consequence of large variance is that the images generated by the network become blurred.

As a deep generative model with solid theoretical foundations, diffusion models have shown great potential in image synthesis and translation. We compared U-Frame with the diffusion model in all our tasks and found that U-Frame outperforms the diffusion model in all these tasks and U-Frame is much more advantageous in showing detailed nuclear information and performs better in the clinical evaluation of the blinded evaluation of virtually stained images by histopathology experts. Meanwhile, the performance of U-Frame could be further improved by replacing the encoder and decoder with more complex structures, which is one of the future research directions. In addition, when the images generated by deep generative models are used for medical purposes, the inference time is important. However, a large number of refinement steps are required for the diffusion model to generate an image, which results in significantly slow inference. In our tests, the inference time of U-Frame was about 2,500 times shorter than that of the diffusion model. For an image with 1,000 × 1,000 pixels, it takes ∼0.3 s for U-Frame and ∼13 min for the diffusion model to generate their respective images.

When training with well-paired datasets, the optimal tolerance size could be so small that there are too many learnable parameters in the network due to the increased number of regions in U-Frame. As a result, physical memory may not be sufficient to accommodate the network. In view of this problem, a lower bound should be added to the automatic strategy for optimizing the tolerance size to reduce the memory requirement. Another way to break the memory limit is that we can design the shape of regions to be irregular and nonlinear by splitting them and controlling the complexity in the hidden feature space, such that the number of parameters is not limited by the original number of regions in U-Frame.

Recent advances in computer vision have revealed that the relationship between image patches plays a crucial role to achieve high performance in visual tasks (38–40). For example, a vision transformer (39) divides the input image into multiple patches, and then the attention is calculated between any two patches to generate effective feature representations for subsequent visual tasks. However, such patches are totally different from the concept of regions in U-Frame. Firstly, a patch is a subset of the input image, while in U-Frame, an input image is a subset of a region. Consequently, algorithms such as convolutions or attention modules can be used for patches but cannot be used directly for regions. Secondly, for each region in U-Frame, there is a unique node in the last layer of discriminators which represents its class information and is to be trained for better exploration of the details in the training images. Third, a patch is usually in square shape but a region in U-Frame can be any nonlinear shape, as long as they are partitioning, i.e. a collection of disjoint subsets of the raw image, which could be useful for memory control as mentioned above during the training of U-Frame.

Although U-Frame can deal with various types of imaging data in microscopic image translation, it may still fail if there is a huge difference between the testing and training data. In such a case, we could try to increase the sample size for training if possible, and minimize the difference during data pre-processing before training and inference. For example, we should scale the testing images so that the size of the nuclei is close to that in the training images, and adjust the contrast as well to improve signal consistency.

In summary, U-Frame bridges unsupervised and supervised learning in a general framework for microscopic image translation. The results show that U-Frame consistently performs the best not only in general simple cases (e.g. fully registered image pairs) but also in cases when both supervised and unsupervised learning become unsuitable. This method is easy to understand and accessible to the scientific community, showing great potential to be a new standard method for microscopic image translation, along with its downstream applications such as clinical diagnosis and image segmentation.

## Materials and methods

### Confocal and widefield image acquisition

The cross-section of a vascular plant was mounted on a glass slide and stained with safranine, which is a fluorescent stain for visualizing lignified tissues. The fluorescence images were acquired using a Nikon C2+ microscope equipped with both confocal and widefield settings. Images were captured with a 60×/1.4 NA objective lens using a laser with an excitation wavelength of 488 nm, and a FITC filter set (excitation: 480/30 nm; emission: 535/40 nm).

### Histology sample preparation and image acquisition

The breast and lung cancer tissues were either surgically excised or through tissue biopsy. The tissues were formalin-fixed and paraffin-embedded. Thin tissue slices, with a thickness of 4 µm, were sectioned and placed on a quartz slide. The tissue slices were deparaffined prior to imaging. The autofluorescence images were acquired using a widefield inverted microscope equipped with a 10×/0.3 numerical aperture (NA) objective lens (Plan Fluorite, Olympus Corp.), an infinity-corrected tube lens (TTL-180-A, Thorlabs Inc.), and a monochrome scientific complementary metal-oxide-semiconductor camera (pco.panda 4.2, PCO Inc.). A deep ultraviolet light-emitting diode of 265 nm (M265L4, Thorlabs Inc.) was used as an excitation light source because of its high absorption in cell nuclei (40, 52), consequently providing high nuclear contrast without labels (53). After acquiring the autofluorescence image, the same slide was stained with H&E, and its brightfield images were captured using a whole-slide scanner equipped with a 20×/0.75 NA objective lens (NanoZoomer-SQ, Hamamatsu Photonics K.K). All human experiments were carried out in conformity with a clinical research ethics review approved by the Institutional Review Board of the Chinese University of Hong Kong/New Territories East Cluster (reference number: 2021.597). Please note that the human samples used in our study are deidentified prior to use.

### Blinded evaluation of virtually stained images by histological experts

We quantify the results of image translation by PSNR and MS-SSIM, a multiscale version of SSIM (44) that incorporates image details at different resolutions. Blinded evaluations were used to assess the clinical value of different virtual staining approaches for cancer diagnosis with human breast biopsy tissue. Specifically, two pathologists were blinded to virtual staining approaches while ground truth is given for references. They were asked to give scores using three types of evaluations: stain quality, morphology quality, and cell-type classification. For stain quality, there are three evaluation parameters including hematoxylin quality, eosin quality, and overall stain quality. These definitions are summarized in Table S4. For morphology quality, there are six evaluation parameters including nuclear circularity, nuclear density, nuclear size, stroma details, and gland morphology. These definitions are summarized in Table S5. The same scale from 0 to 5 was applied to the scores of stain quality and morphology quality: 5, excellent (very high agreement); 4, very good (high agreement with minor differences); 3, good (moderate agreement); 2, acceptable (low agreement); 1, unacceptable (very high disagreement); 0, cannot show/cannot determine. For cell-type classification, cancer cells and lymphocytes are differentiated, and the results were quantitatively evaluated by Intersection over Union (54) and Dice scores (55), which were calculated based on the binary mask of cancer cells.

### Global sampling rule in U-Frame

The discriminators of U-Frame are multiclass classifiers, where each class represents a region with unique feature patterns in the image. In practice, however, many regions in different classes could be similar. As a result, a sampling rule with uniform probability can lead to low performance due to imbalanced training of the feature patterns in the image. Therefore, a global sampling rule is used to balance such an effect (Fig. S1). In each iteration, the training patch as input is selected according to the probability determined by


$$Q_{i}=\frac{1+\frac{\mathrm{cov}(S_{i},T_{i})}{\sigma_{S_{i}}\sigma_{T_{i}}}}{\sqrt{\sum_{\;j=1}^{N}\;H_{i}\times H_{j}}}\;$$

where $\sigma_{s_{i}}$ and $\sigma_{T_{i}}$ are the SDs of the source image ($S_{i}$) and target image ($T_{i}$), respectively, cov is the covariance, $H_{i}$ is a vector of the normalized image histogram for region *i*, and *N* is the total number of exclusive regions. $Q_{i}$ consists of a Pearson correlation coefficient between the source image and target image in the numerator, such that the higher the similarity between the two domains, the higher the probability of the sampling. The denominator is the dot product of pixel distribution between the selected region and other regions, such that the lower the similarity (i.e. the lower the occurrence), the higher the sampling probability should be given for training the rare and special regions. The probability of the selected region being trained in the current iteration is a scaled result from $Q_{i}$, given by


$$P_{i}=\frac{Q_{i}}{\sum_{\;j=1}^{N}\;Q_{j}}$$

### Network architecture and loss function

The network structure of U-Frame is an extension of basic GAN architecture with standard convolutional neural networks. The network of U-Frame is composed of two generators and two discriminators. Given one training sample ($S_{i}$, $T_{i}$), one generator transforms $S_{i}$ into $T_{i}$, and the other generator transforms $T_{i}$ into $S_{i}$. Each generator consists of an encoder projecting the input into a latent space, which is shared between the generators, and a decoder converting from the latent space to the original space.

A reconstruction loss is calculated by the least absolute deviations between the input and decoded image from the same generator:


$$L_{\mathrm{rec}}=\underset{i}{\sum }\;|S_{i}-U_{S}(E_{S}(S_{i}))|+\underset{i}{\sum }\;|T_{i}-U_{T}(E_{T}(T_{i}))|$$

where $E_{S}$ and $U_{S}$ are the encoder and decoder of the source image domain, respectively, $E_{T}$ and $U_{T}$ are the encoder and decoder of the target image domain, respectively.

The discriminators of U-Frame not only check whether an image is real or fake but also check which region it comes from if it is real, which can be included in the loss function as


$$\begin{array}{rl} CE_{d}\;= & \;\;\;-\underset{i}{\sum }\;Y_{i}\;\ln(D_{T}(T_{i}))-\underset{i}{\sum }\;Y_{i}\;\ln(D_{S}(S_{i}))-Y_{0}\underset{i}{\sum }\;\ln(D_{T}(U_{T}(E_{S}(S_{i}))))\\ & \;-Y_{0}\underset{i}{\sum }\;\ln(D_{S}(U_{S}(E_{T}(T_{i})))) \end{array}$$

where $CE_{d}$ is the cross-entropy loss for updating the discriminator in an iteration. $D_{S}$ and $D_{T}$ are the discriminators, $Y_{i}$ and $Y_{0}$ are one-hot encoded labels for region *i* and fake image, respectively.

The cross-entropy loss for updating the generator in an iteration is defined as


$$CE_{g}=-\underset{i}{\sum }\;Y_{i}\;\ln(D_{T}(U_{T}(E_{S}(S_{i}))))-\underset{i}{\sum }\;Y_{i}\;\ln(D_{S}(U_{S}(E_{T}(T_{i}))))$$

Except for the cross-entropy, the network also includes a cycle-consistency loss, which is defined as the sum of the forward consistency from source to target image, and backward consistency from target to source image:


$$L_{\mathrm{cycle}}=\underset{i}{\sum }\;|S_{i}-U_{S}(E_{T}(U_{T}(E_{S}(S_{i}))))|+\underset{i}{\sum }\;|T_{i}-U_{T}(E_{S}(U_{S}(E_{T}(T_{i}))))|$$

The generators and discriminators are optimized alternatively with different loss functions. We define the loss function for the generators as


$$L_{g}=CE_{g}+\lambda_{1}L_{\mathrm{cycle}}+\lambda_{2}L_{\mathrm{rec}}$$

where $\lambda_{1}$ and $\lambda_{2}$ are the weights for the cycle-consistency loss and reconstruction loss, respectively. The loss function for the discriminators is defined as


$$L_{d}=CE_{d}$$

In the last layer of the discriminator, there are *N* + 1 nodes representing *N* exclusive regions and one fake class for the images generated by the generators.

### Training and implementation details

For the preparation of virtual histological staining, raw autofluorescence images for virtual staining are first stitched using a grid stitching plugin in ImageJ (56). The autofluorescence and the corresponding histochemically stained images are coarsely registered by estimating the optimal transform based on the corresponding points on the two images. Otsu's method (57) is first used to obtain a threshold to estimate the range of background noise values and the pixels below the threshold are recorded for further analysis. Since the distribution of noise should be dominant, an interquartile range method (58) is then used to estimate the outlier. The top 0.1% of the pixel values are saturated and the remaining pixel values are linearly scaled accordingly.

For data requiring registration, global registration was performed on the raw images by affine mapping to correct slight shifts, scaling, or rotations. After initialization by global registration, local registration was used to match the source and target images accurately. For patches cropped from the source image, the corresponding matched patches in the target image were optimized by local affine mapping with the loss function of Pearson correlation coefficient.

When the source images have one channel only and the target images have three channels, we calculate the two derivatives of the Sobel–Feldman operator (59). The derivatives are scaled and converted to unsigned 8-bit types as additional two channels, which are then concatenated with the original channel as the input of networks.

All the models in this study were trained from scratch, without pretraining on other datasets. For U-Frame in this study, we set the weights of the loss function as $\lambda_{1}=10$, $\lambda_{2}=10$, $\lambda_{3}=0.01$. The overlapping size of U-Frame was optimized and set as 64 (Table S6). The weights of discriminators were randomly initialized by a normal distribution with a mean of 0 and SD of 0.02, and the weights of generators were initialized by Kaiming initialization with preserving the magnitude of the variance of weights in the forward pass (60). All the biases were initialized as 0. The learnable parameters were updated by the Adam optimizer (61). We set the total number of epochs as 80. The learning rate was fixed as 0.0002 for epochs of 0 to 40, and then linearly decayed from 0.0002 to 0 for epochs of 41 to 80. The batch size was set as 2. For pix2pix, generators with residual blocks of 10 layers were used. The weight of L1 loss for pix2pix was set as 100. The optimizer, learning rate schedule, training epoch, and batch size were all the same as that for U-Frame. For CycleGAN, the weights for cycle loss and identity loss were set as 10 and 0.5, respectively. The optimizer, learning rate schedule, training epoch, and batch size were all the same as that for U-Frame. For the diffusion model, we used the network as proposed in Ref. (34). Specifically, denoising diffusion probabilistic models (32) were used as network structures. Additional conditioning of the source image was implemented via concatenation in the backward process as suggested (33). During training, we used a linear noise schedule of (1*e*^−6^, 0.01) with 2,000 time-steps, and used 1,000 refinement steps with a linear schedule of (1*e*^−4^, 0.09) during inference. For CARE, the default setting was used as in reference (16). To compute the confidence intervals of MS-SSIM and PSNR, the corresponding experiment was repeated 5 times keeping all the settings exactly the same.

The network was implemented with Pytorch version 1.8.1 (62) and Python version 3.8.8 in the Ubuntu 20.04.1 long-term support operating system. The training was performed on the computer equipped with 35 Core i9-10980XE CPUs at 3.0 GHz (Intel), 256 GB of RAM, and 4 GeForce RTX 3090 GPUs (Nvidia).

## Supplementary Material

pgae133_Supplementary_Data

## Data Availability

All data involved in this work including raw/processed images provided in the manuscript and supplementary information, and the source code of U-Frame are available at https://github.com/TABLAB-HKUST/U-Frame.

## Preprints

This manuscript was posted on a preprint: https://doi.org/10.36227/techrxiv.19747057.v1g.
