# Supplementary material for: Exceeding the limit for microscopic image translation with a deep learning-based unified framework
Source: PNAS Nexus. 2024 Mar 29;3(4):pgae133. doi: 10.1093/pnasnexus/pgae133 (PMC11004937; doi:10.1093/pnasnexus/pgae133)
Supplement: pgae133_Supplementary_Data [file pgae133_supplementary_data.docx]

**
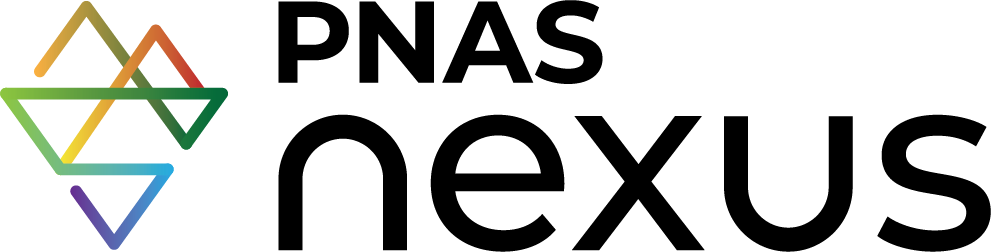
**

**Supplementary Information for**

Exceeding the limit for microscopic image translation with a deep learning-based unified framework

Weixing Dai, Ivy H. M. Wong, Terence T. W. Wong*

Translational and Advanced Bioimaging Laboratory, Department of Chemical and Biological Engineering, Hong Kong University of Science and Technology, Hong Kong, China.

* Terence T. W. Wong

**Email:** ttwwong@ust.hk

**This PDF file includes:**

Figures S1 to S8

Tables S1 to S6


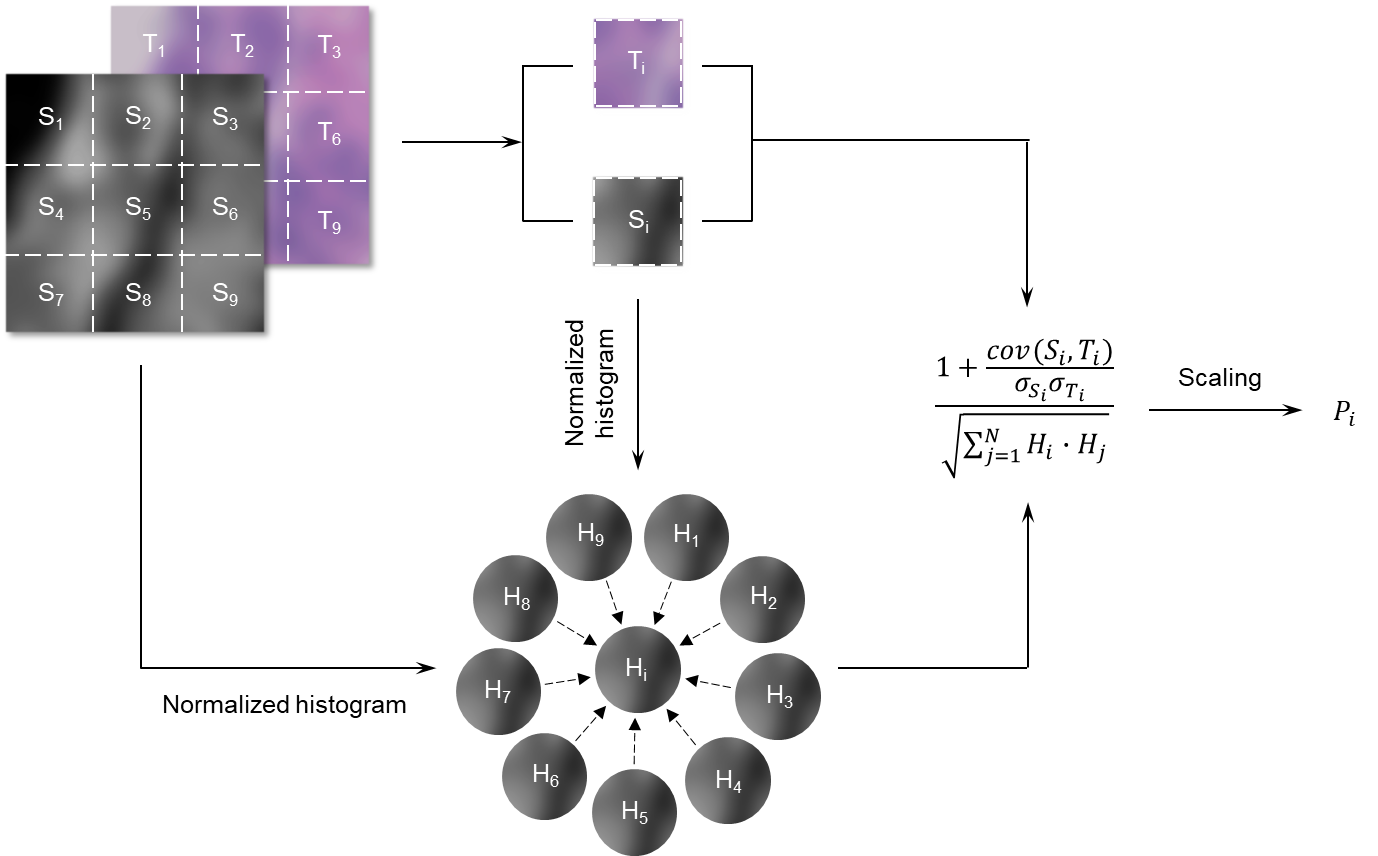


**Fig. S1.** Global sampling rule implemented in U-Frame. Given a selected region $\left( S_{i},T_{i} \right)$, the probability of being trained in the current iteration $P_{i}$ is calculated according to the global sampling rule as illustrated. $S_{i}$ and $T_{i}$are selected regions from the source and target images, respectively. $H_{i}$ is the normalized histogram of the selected region from the source image. $\sigma_{s_{i}}$ and $\sigma_{T_{i}}$ are the standard deviations of $S_{i}$and $T_{i}$, respectively, and $cov$ is the covariance.


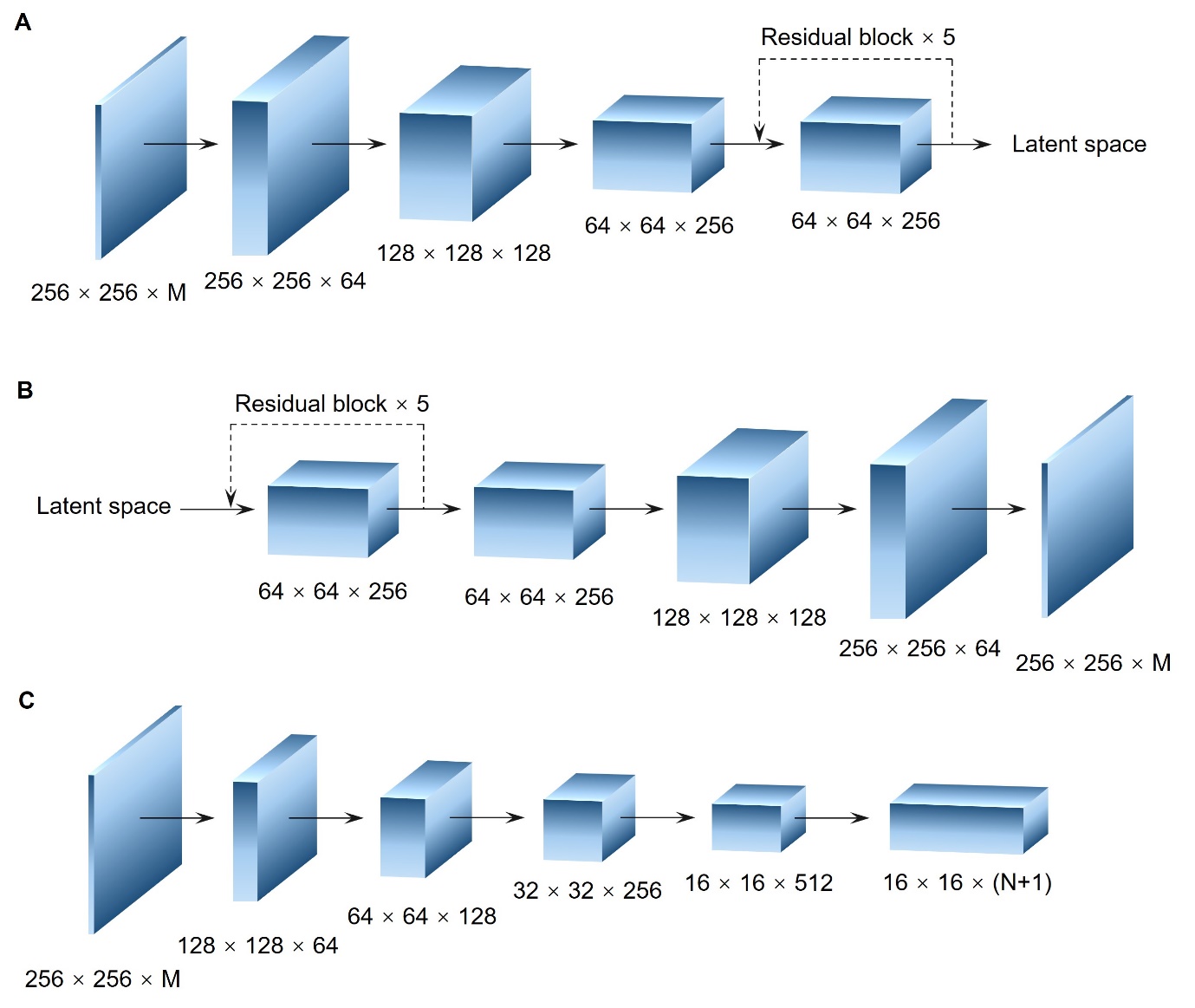


**Fig. S2**. **Network architecture of U-Frame.** (A) Architecture of the encoder. The encoder transforms the input image into a latent space. M is the number of input channels. (B) Architecture of the decoder. The decoder transforms the latent space representation into a generated image. (C) Architecture of the discriminator. The discriminator not only classifies real images from the images generated by the generator, but also distinguishes the real images in different spatial locations that show various patterns from each other. N is the total number of exclusive regions.

**
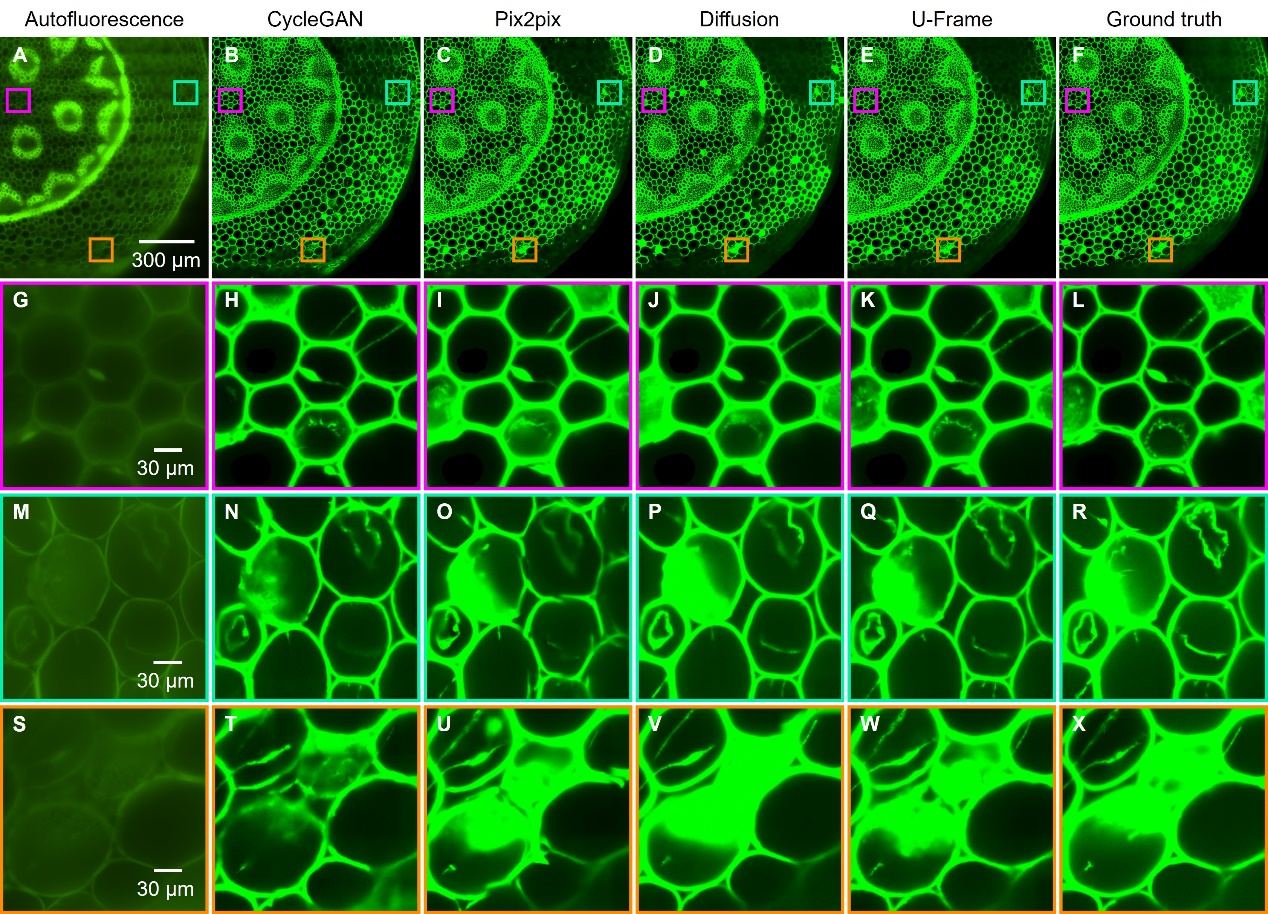
**

**Fig. S3. From widefield to confocal image transformation.** (A) Widefield image in the test set. (B–E) Image transformed by CycleGAN, pix2pix, diffusion model, and U-Frame, respectively. (F) Confocal image of (A) at a single depth. (G–L) Zoomed-in regions of magenta squares marked in (A–F), respectively. (M–R) Zoomed-in regions of cyan squares marked in (A–F), respectively. (S–X) Zoomed-in regions of orange solid squares marked in (A–F), respectively.


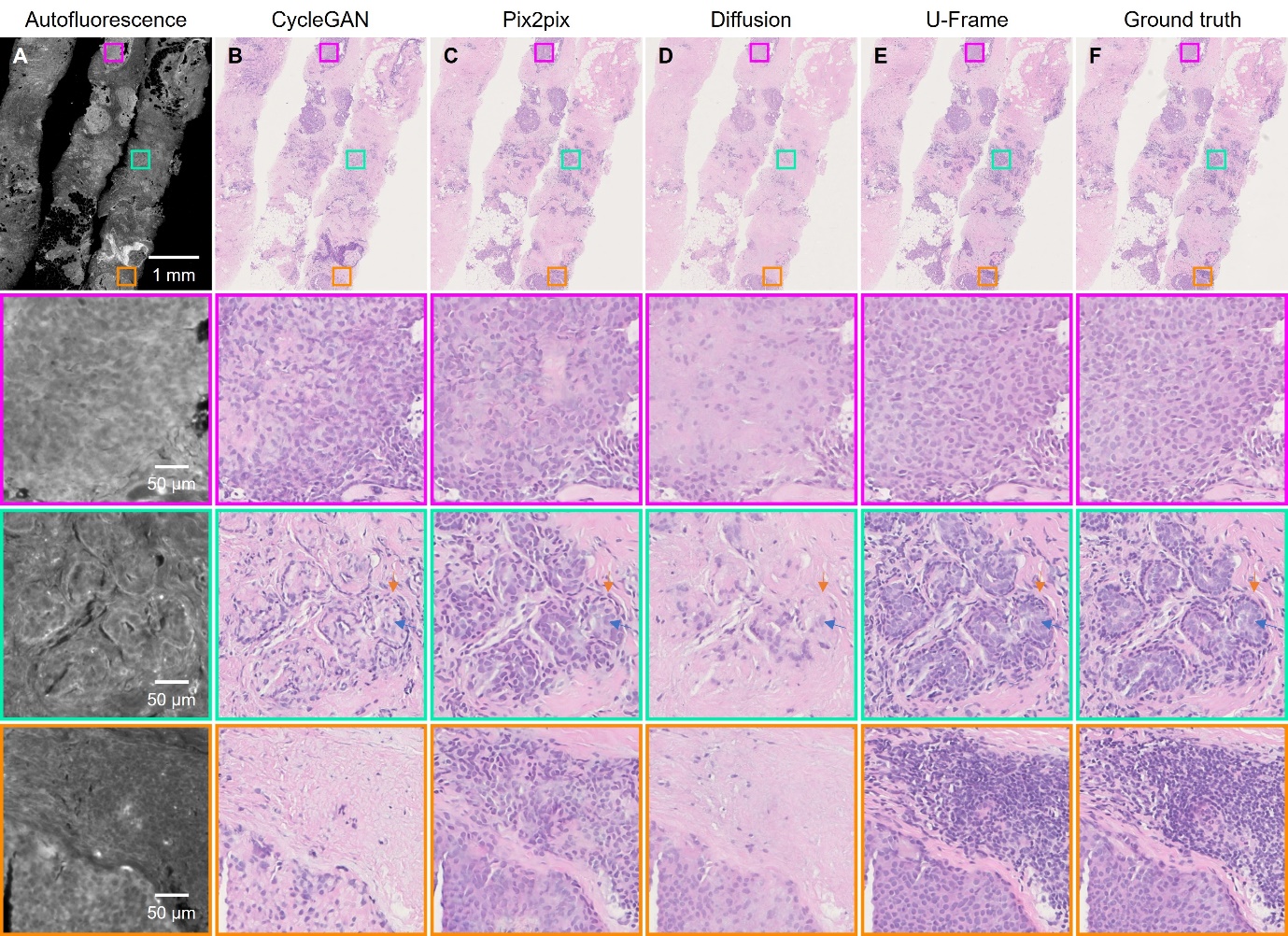


**Fig. S4. H&E virtual staining of breast cancer tissue (dataset I).** In this dataset, autofluorescence and H&E images are from the same tissue slice so that they can be well aligned. (A) Autofluorescence image of a human breast tissue biopsy. (B**–**E) Virtual H&E-stained images transformed from (A) by CycleGAN, pix2pix, diffusion model, and U-Frame, respectively. (F) Real H&E-stained image of (A) as the ground truth. The zoomed-in regions of magenta, cyan, and orange squares marked in (A–F) are shown below their respective original images.


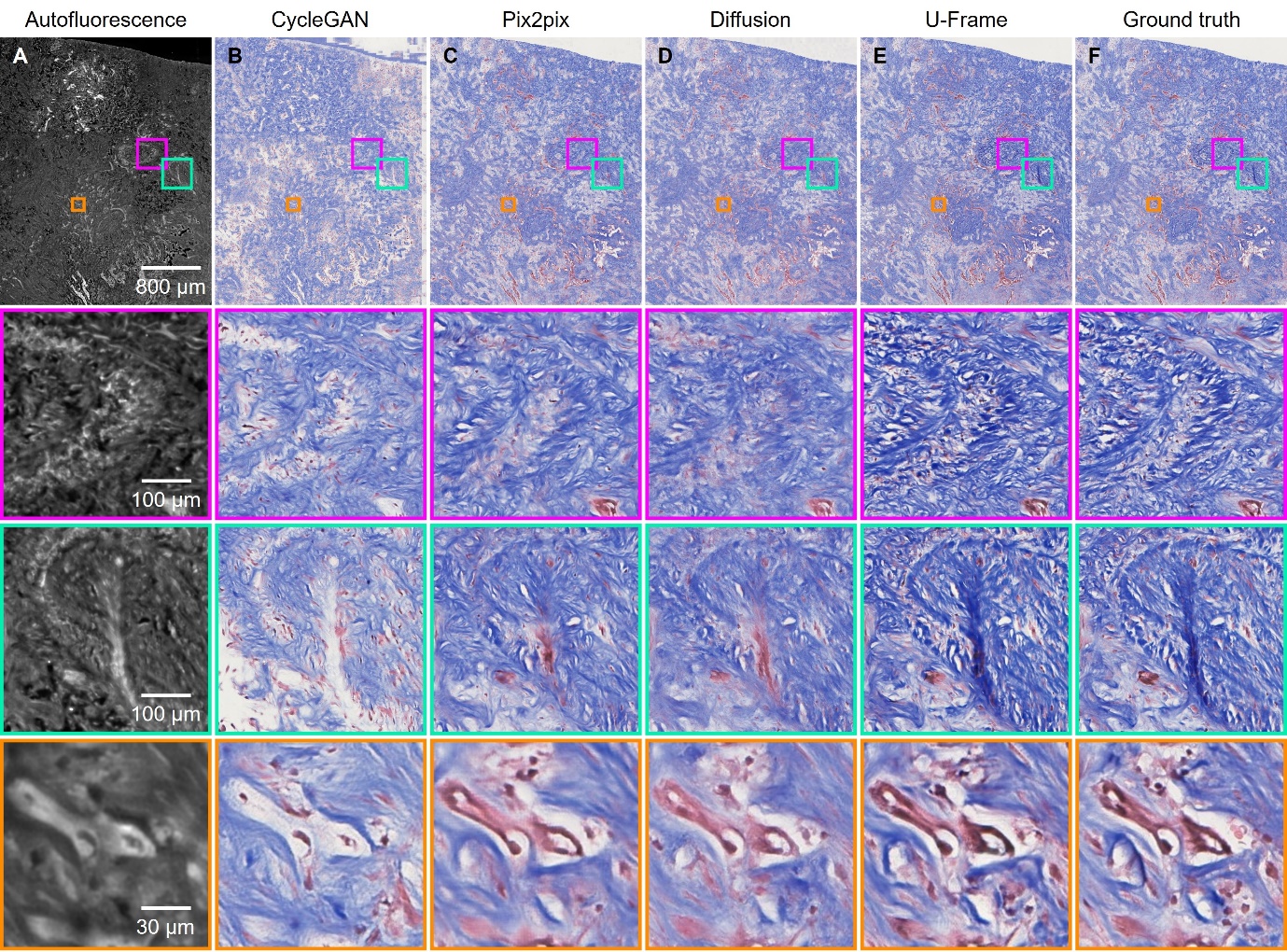


**Fig. S5. Masson’s trichome virtual staining of liver cancer tissue.** (A) Autofluorescence image of a human liver cancer tissue. (B–E) Virtual Masson’s trichome-stained images transformed from (A) by CycleGAN, pix2pix, diffusion model, and U-Frame, respectively. (F) Real Masson’s trichome-stained image of (A) as the ground truth. The zoomed-in regions of magenta, cyan, and orange squares marked in (A–F) are shown below their respective original images.


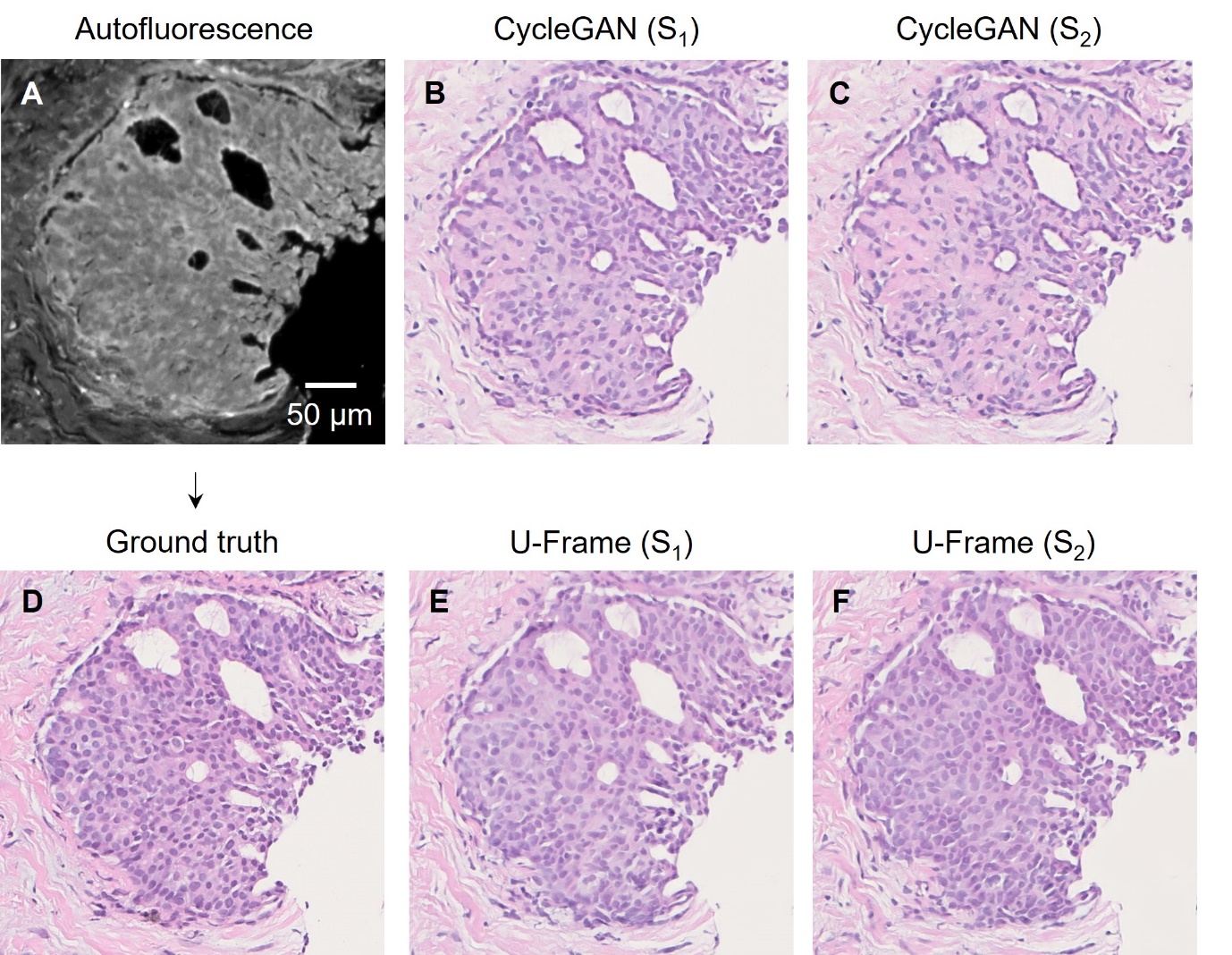


**Fig. S6. Performance tests on datasets with two different levels of spatial misalignment of the input data.** (A) Autofluorescence image of the testing set. (B) Performance of CycleGAN trained on dataset S_1_, where the data were roughly aligned. (C) Performance of CycleGAN trained on dataset S_2_, where the data were generated by randomly pairing the patches of source and target images that are 256 pixels away from each other. (D) The corresponding real H&E-stained image serves as the ground truth. (E) Performance of U-Frame trained on dataset S_1_. (F) Performance of U-Frame trained on dataset S_2_.


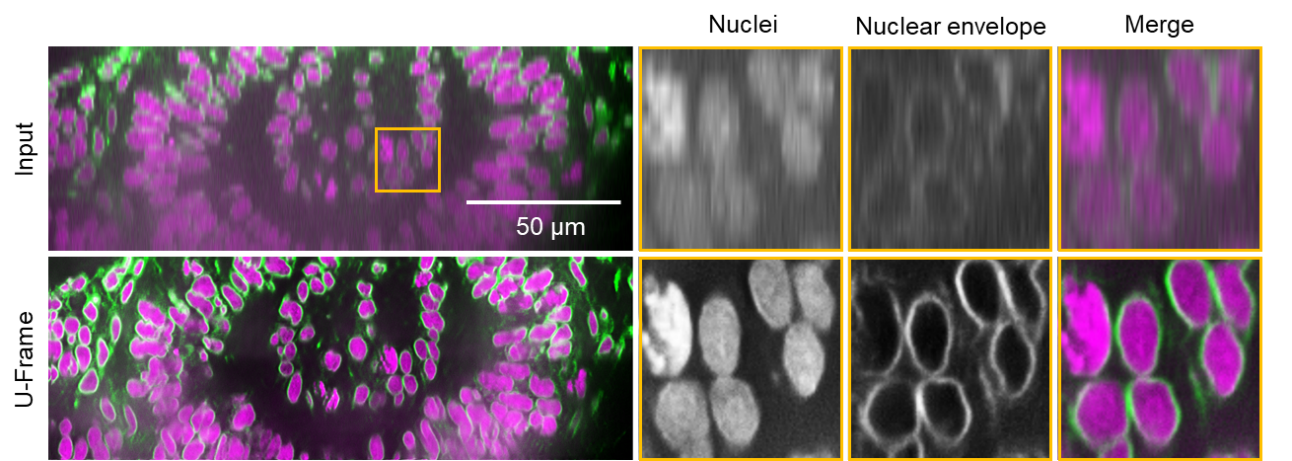


**Fig. S7. Image resolution recovery from anisotropic images of the zebrafish retina.** The nuclei and the nuclear envelope were digitally stained as magenta and green, respectively.


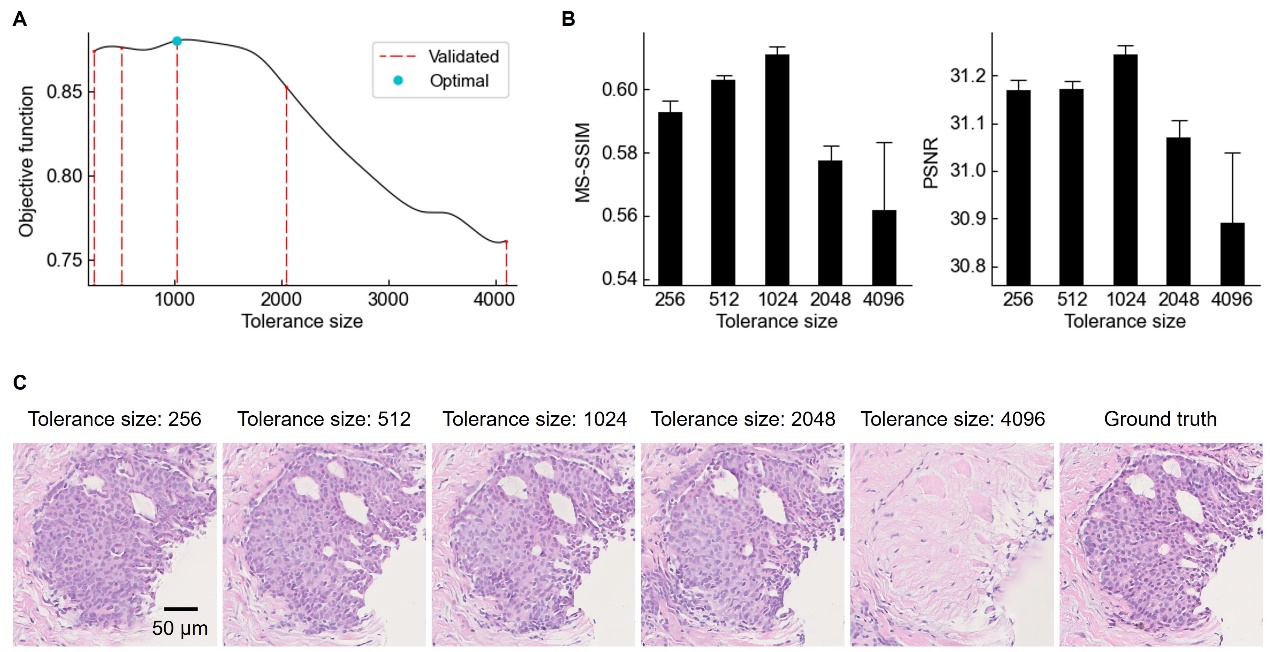


**Fig. S8. Validation of the strategy for automatically selecting tolerance size.** The dataset used here was the breast cancer dataset (S_2_) where a random shift of 256 pixels was introduced. (A) The curve of the objective function for the strategy of automatically selecting tolerance size, (B) Quantitative metrics (MS-SSIM and PSNR) for the validation of the strategy. (C) Transformed images by U-Frame with different tolerance sizes for the validation of the strategy.

**Table S1.** Details of the encoder for U-Frame.

| Layer | Output size | Kernel size | Stride | Padding | Activation |
| --- | --- | --- | --- | --- | --- |
| Conv | 256 × 256 × 64 | 7 × 7 | 1 | 3 | ReLU |
| Conv | 128 × 128 × 128 | 4 × 4 | 2 | 1 | ReLU |
| Conv | 64 × 64 × 256 | 4 × 4 | 2 | 1 | ReLU |
| ResNet × 5 | 64 × 64 × 256 | 3 × 3 | 1 | 1 | ReLU |
|  | 64 × 64 × 256 | 3 × 3 | 1 | 1 | None |

**Table S2.** Details of the decoder for U-Frame.

| Layer | Output size | Kernel size | Stride | Padding | Activation |
| --- | --- | --- | --- | --- | --- |
| ResNet × 5 | 64 × 64 × 256 | 3 × 3 | 1 | 1 | ReLU |
|  | 64 × 64 × 256 | 3 × 3 | 1 | 1 | None |
| Upsample | 128 × 128 × 256 | None | None | None | None |
| Conv | 128 × 128 × 128 | 5 × 5 | 1 | 2 | ReLU |
| Upsample | 256 × 256 × 128 | None | None | None | None |
| Conv | 256 × 256 × 64 | 5 × 5 | 1 | 2 | ReLU |
| Conv | 256 × 256 × 3 | 7 × 7 | 1 | 3 | Tanh |

**Table S3.** Details of the discriminator for U-Frame.

| Layer | Output size | Kernel size | Stride | Padding | Activation |
| --- | --- | --- | --- | --- | --- |
| Conv | 128 × 128 × 64 | 4 × 4 | 2 | 1 | Leaky ReLU |
| Conv | 64 × 64 × 128 | 4 × 4 | 2 | 1 | Leaky ReLU |
| Conv | 32 × 32 × 256 | 4 × 4 | 2 | 1 | Leaky ReLU |
| Conv | 16 × 16 × 512 | 4 × 4 | 1 | 1 | Leaky ReLU |
| Conv | 16 × 16 × (*N* + 1) | 1 × 1 | 1 | 0 | None |

**Table S4.** Definition of evaluation parameters for stain quality in the clinical evaluation of stained images.

| Parameter | Definition |
| --- | --- |
| Hematoxylin quality | Degree of color tone agreement for hematoxylin content compared with ground truth |
| Eosin quality | Degree of color tone agreement for eosin content compared with ground truth |
| Overall stain quality | Overall color tone agreement compared with ground truth |

**Table S5.** Definition of evaluation parameters for morphology quality in the clinical evaluation of stained images.

| Parameter | Definition |
| --- | --- |
| Nuclear circularity | Degree of circularity (shape) agreement of nuclei compared with ground truth |
| Nuclear density | Degree of density agreement of nuclei compared with ground truth |
| Nuclear size | Degree of nuclear size agreement compared with ground truth |
| Stroma details | Degree of stroma details (e.g., orientation, density, thickness) agreement compared with ground truth |
| Gland morphology | Degree of similarity of gland architecture compared with ground truth |

**Table S6.** An ablation study of U-Frame with different configurations of global sampling rule and overlapping size.

| Index | Overlapping size | Global sampling rule | MS-SSIM |
| --- | --- | --- | --- |
| 1 | 0 | ✓ | 0.6135 |
| 2 | 4 | ✓ | 0.6126 |
| 3 | 8 | ✓ | 0.6122 |
| 4 | 16 | ✓ | 0.6103 |
| 5 | 32 | ✓ | 0.6213 |
| 6 | 64 | ✓ | **0.6274** |
| 7 | 128 | ✓ | 0.6135 |
| 8 | 64 | × | 0.6104 |
